# Low Visual Acuity Among Children in Public Schools in the Northeast of Brazil: A Cross-Sectional Study

**DOI:** 10.7759/cureus.85349

**Published:** 2025-06-04

**Authors:** Lucas Neves de Oliveira, Matheus Gomes Reis Costa, Isadora Oliveira Santiago Pereira, Isabele Carolina Tokumoto, Joao Lucas de Magalhães Leal Moreira, Matheus Carneiro Leal Freitas, Clarissa Silva Sampaio, Mateus Neves de Oliveira, Jose Bessa Junior, Hermelino Lopes de Oliveira Neto

**Affiliations:** 1 Faculty of Medicine, State University of Feira de Santana, Feira de Santana, BRA; 2 Faculty of Medicine, Bahian School of Medicine and Public Health, Salvador, BRA; 3 Department of Public Health, State University of Feira de Santana, Feira de Santana, BRA; 4 Department of Ophthalmology, State University of Feira de Santana, Feira de Santana, BRA

**Keywords:** prevalence, public health, students, vision disorders, visual acuity, cross-sectional studies

## Abstract

Objective

This study describes the prevalence of low visual acuity (LVA) in public school students from a city in the interior of the Northeast region of Brazil.

Methods

This was an observational, cross-sectional, exploratory study. The sample consisted of schoolchildren from the second to the fourth grade of five public schools in Feira de Santana, Bahia, Brazil. Data collection was carried out in the schools themselves, with a sociodemographic and clinical questionnaire applied and visual acuity (VA) measured using the Snellen “E” optotype chart. LVA was defined as uncorrected VA < 20/25 in at least one eye.

Results

The sample consisted of 358 children, with a median age of nine (IQR 8-10) years, of which 189 (52.9%) were female, and 169 (47.2%) were male. A total of 248 (69.3%) individuals had never been to an ophthalmologist. LVA was found in 105 (29.3%) schoolchildren, and of these, 7.6% (8/105) currently used glasses. Factors associated with LVA were female gender and white ethnicity. LVA was evidenced in 60 (31.7%) schoolchildren with excessive screen use and in 35 (25.5%) without excessive use (OR 1.35; 95% CI 0.83-2.19, p = 0.222), and excessive screen use was associated with visual signs/symptoms such as tearing and eye itching.

Conclusion

LVA was observed in approximately 30% (n = 105) of children in public schools in the interior of Bahia, and less than 10% of these children used glasses. Our study reinforces the importance of visual screening of schoolchildren through active search in our region and the creation of strategies to facilitate access to ophthalmological consultations and glasses.

## Introduction

Vision, among the five senses, is the most dominant and the primary means of integrating the individual with the external environment, with a large part of knowledge being acquired visually [1]. Visual problems impair learning and social interactions, compromising intellectual, academic, and professional development, as well as communication and socialization skills [2].

School-aged children are particularly affected by vision impairment. Initially, visual problems may not be perceived by the family, mainly due to the absence of signs or complaints. Over time, significant visual effort becomes evident in the teaching-learning process [3]. If persistent, these problems affect the child’s academic performance and socialization [4].

According to the World Health Organization, there are approximately 1.4 million children with visual impairment worldwide, with 90% living in developing countries. Each year, 500,000 children become blind, and about 80% of childhood blindness causes are preventable or treatable [1]. It is estimated that the prevalence of childhood blindness in Brazil is 4/10,000 children [5]. Concerning reversible blindness, the leading cause of childhood blindness, uncorrected refractive errors are the primary cause of low vision in school-aged children [1].

In Brazil, there is limited data on the prevalence of visual impairment among schoolchildren, and to our knowledge, no data are available for Bahia. Additionally, many studies are outdated, and most were conducted in the South and Southeast regions. A study conducted in Sorocaba, São Paulo, showed a prevalence of low visual acuity (LVA) of 13.1% in public school children [6]. In Londrina, Paraná, the prevalence of LVA was demonstrated in 17.1% of public school students [7]. In Patos de Minas, Minas Gerais, the prevalence of visual impairment in schoolchildren was 20.9% [8].

Early diagnosis of visual disorders has been suggested as a strategy to prevent future problems, including amblyopia, and alterations in neuropsychomotor and social development [9]. From a public health perspective, routine visual acuity (VA) assessment is essential for promoting eye health, contributing to reducing high levels of school dropout and poor academic performance [6,10].

Given the importance of early diagnosis, the scarcity of data in national literature, especially in the Northeast, and the absence of studies in Bahia, the aim of this study was to evaluate the prevalence of LVA in public school children in Feira de Santana, Bahia. An initial version of this article was previously published on a preprint server [11].

## Materials and methods

Study population

We conducted a cross-sectional study between August 2022 and May 2023. The study participants were elementary school students from the second to the fourth grade, regularly enrolled in five municipal public schools located in Feira de Santana, Bahia, Brazil. To ensure a representative and randomized sample, we first obtained a list of all municipal public schools from the Municipal Health Department. Using a random number generator, we randomly selected five schools located in socioeconomically diverse areas of the city.

This observational study was approved by the Research Ethics Committee of the State University of Feira de Santana on May 1, 2022, under protocol no. 56993722.5.0000.0053, position statement 5.380.168. Written informed consent was obtained from the parents or guardians of the children.

Data collection

Data were collected at the schools by medical students from the Visual Disorders Combat League (LCDV) of the State University of Feira de Santana (UEFS), adequately trained by ophthalmologists at an eye hospital. Initially, a sociodemographic and clinical questionnaire was administered to parents/guardians, including reports of recurring ophthalmic signs/symptoms and excessive screen use.

VA was assessed using the Snellen "E" optotype chart, positioned 6 m away at a height of 1.5 m. All children were first evaluated without optical correction; for those who already used glasses, the test was repeated with their habitual correction [7,12-14].

Definitions and classifications

VA was classified as normal vision (VA ≥ 20/25 or 0.8), mild visual impairment (VA < 20/25 or 0.8 and ≥ 20/63 or 0.3), moderate visual impairment (VA < 20/63 or 0.3 and ≥ 20/160 or 0.125), and severe/profound visual impairment (VA < 20/160 or 0.125 and ≥ 20/1000 or 0.02) [14,15]. LVA was defined as uncorrected VA < 20/25 in at least one eye [6-8,16].

Excessive screen use was defined as greater than four hours per day, based on the parent-reported single-question assessment. Screens included are cell phones, tablets, computers, and televisions.

Statistical analysis

Data are presented as absolute values, frequencies, medians, and interquartile ranges (IQRs). Categorical variables were compared using the chi-square test, and the odds ratio (OR) was used as a measure of association for categorical variables. A p-value < 0.05 was considered statistically significant, and a 95% confidence interval (CI) was presented as a measure of precision. Statistical analyses were performed using GraphPad Prism version 10.2.2 (GraphPad Software Inc., San Diego, CA).

## Results

Of the initial 361 children enrolled, 358 were included in the final analysis. One was excluded due to a developmental disorder, and two refused to participate. The median age was nine (IQR 8-10) years, and 189 (52.8%) were female. A total of 311 (86.9%) self-identified as Black/Brown, and 241 (67.3%) had a family income ≤ 1 minimum wage. A total of 248 (69.3%) children had never visited an ophthalmologist.

Thirty (8.4%) children reported previous or current use of glasses. Of these, 10 (33%) were current users, and the other 20 (67%) had stopped using them. The sample characteristics are detailed in Table 1.

**Table 1:** Baseline characteristics of the study participants (n = 358) n: number

Baseline characteristics	n (%)
Gender	
Male	169 (47.2)
Female	189 (52.8)
Grade	
2nd	136 (38)
3rd	119 (33.2)
4th	103 (28.8)
Race (self-reported)	
Black	106 (29.6)
Brown	205 (57.3)
White	39 (10.9)
Yellow	4 (1.1)
Indigenous	4 (1.1)
Family income	
≥3 minimum wage	13 (3)
Between 1 and 3 minimum wages	104 (29.1)
≤1 minimum wage	241 (67.3)
Residence	
Urban area	331 (92.5)
Rural area	27 (7.5)
Prematurity	
No	323 (90.2)
32 to 36 weeks	30 (8.4)
28 to 31 weeks	4 (1.1)
≤28 weeks	1 (0.3)
Systemic disease	
Total	52 (14.5)
Respiratory diseases	28 (7.8)
Rhinitis/sinusitis	14 (3.9)
Asthma	13 (3.6)
Previous consultation with an ophthalmologist	
No	248 (69.3)
Yes	110 (30.7)
Use of glasses	
Previous or current	
No	328 (91.6)
Yes	30 (8.4)
Current	
No	348 (97.2)
Yes	10 (2.8)

Prevalence of LVA

LVA (uncorrected VA < 20/25 in at least one eye) was found in 105 (29.3%) students. Of these, 7.6% (8/105) were current glasses users, and after correcting VA with glasses, six still had visual impairment in at least one eye. The distribution of VA is detailed in Table 2.

**Table 2:** VA distribution (n = 358) n: number; VA: visual acuity

VA classification	Uncorrected VA	Current glasses users
	n (%)	n (%)
Normal vision (≥20/25), both eyes	253 (70.7)	2 (0.8)
Normal vision (≥20/25), one eye only	29 (8.1)	1 (3.4)
Mild visual impairment (<20/25 and ≥20/63), better eye	56 (15.6)	3 (5.3)
Moderate visual impairment (<20/63 and ≥20/160), better eye	14 (3.9)	2 (14.3)
Severe/profound visual impairment (<20/160 and ≥20/1000), better eye	6 (1.7)	2 (33.3)
Total	358 (100)	10 (2.8)

In the univariate analysis, the variables associated with LVA were female gender (OR 2.12; 95% CI 1.32-3.41, p = 0.002) and white race (OR 2.57; 95% CI 1.31-5.05, p = 0.006). Age, family income, and prematurity were not associated (Table 3).

**Table 3:** Univariate analysis - variables associated with LVA (n = 358) CI: confidence interval; LVA: low visual acuity; n: number; OR: odds ratio

Variables	OR	95% CI	p-value
Female	2.12	1.32-3.41	0.002
Age > 11 years	2.47	0.85-7.24	0.098
White race	2.57	1.31-5.05	0.006
Family income > 1 minimum wage	1.25	0.77-2.02	0.362
Prematurity	1.12	0.53-2.37	0.774

Vision and excessive screen use

In the analysis of these aspects, our sample was reduced to 330 students due to incomplete data in this aspect for 28 subjects.

Excessive screen use was found in 189 (57.3%) children. LVA was observed in 60 (31.7%) students with excessive screen use and in 36 (25.5%) without excessive screen use (OR 1.35; 95% CI 0.83-2.19, p = 0.222).

**Figure 1:**
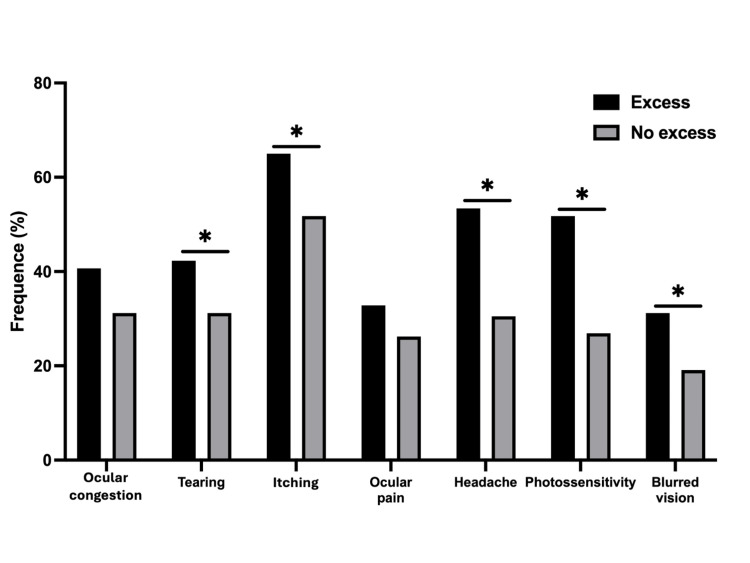
Visual signs/symptoms in schoolers with or without excessive screen use (n = 330) *p < 0.05

Tearing was reported in 40.2% of children with excessive screen use versus 28.4% in the no-excess group (OR 1.62; 95% CI 1.02-2.56; p = 0.040). Itching was significantly more common in the excessive group (56.6% vs. 41.2%; OR 1.73; 95% CI 1.11-2.71; p = 0.015), as were headache (52.4% vs. 29.4%; OR 2.62; 95% CI 1.65-4.14; p < 0.001), photosensitivity (58.7% vs. 30.4%; OR 2.92; 95% CI 1.83-4.66; p < 0.001), and blurred vision (41.3% vs. 26.5%; OR 1.92; 95% CI 1.14-3.22; p = 0.014). However, no statistically significant associations were found for ocular congestion (OR 1.52; 95% CI 0.96-2.40; p = 0.076) or ocular pain (OR 1.37; 95% CI 0.85-2.22; p = 0.199), despite a higher prevalence in the excessive screen use group. These findings are summarized in Figure 1.

## Discussion

The prevalence of LVA in public school children in Feira de Santana, Bahia, was 29.3%. In national literature, the prevalence of LVA in schoolchildren ranged from 9% to 20.87% [6-8,12,14,16]. Our study found a higher prevalence of LVA than previous literature data. We believe several probable factors may explain this significant difference: (1) specific local socioeconomic challenges in public schools in Feira de Santana, Bahia, limiting access to eye care; (2) more sensitive screening methods used in our study, identifying more cases; and (3) differences in sample characteristics compared to previous studies, including age, race, and gender.

The prevalence of current glasses use in this study was 2.8%. In previous studies in Brazil, the prevalence ranged from 2.4% to 4.5% [6,7,12,14,16]. Regarding current glasses use in children with LVA, our study revealed a prevalence of 7.6%, while previous national studies showed a prevalence ranging from 10.52% to 40% [6,8,12,14].

In addition to the higher prevalence of LVA in the studied population, a lower prevalence of current glasses use was demonstrated in children with low vision. It is noteworthy that approximately 70% of the students had never had an ophthalmological consultation. The Brazilian Society of Pediatric Ophthalmology recommends a complete routine ophthalmological consultation from six to twelve months of age and another consultation from three to five years of age, with the frequency of subsequent consultations being determined by the ophthalmologist, usually on an annual basis [17]. The findings reinforce the importance of conducting visual screening actions through active search in our region, as well as creating strategies to facilitate access to ophthalmological consultations and glasses.

The early detection and treatment of visual impairment in infants aim to ensure normal physical and cognitive development. Motor development and communication ability are impaired in infants with visual impairment because gestures and social behaviors are learned through visual feedback [1,5]. There is an additional risk of developing amblyopia, characterized by low vision due to abnormal development of the visual cortex during childhood, which can affect one or both eyes [9,18]. Amblyopia should ideally be treated by the age of seven to eight. Some studies indicate benefit in treatment at older ages, but there is consensus that early correction provides the best prognosis [19].

From a public health perspective, population investigation by ophthalmologists is unfeasible and costly, making routine visual screening by adequately trained non-medical personnel essential [5,20].

A low-cost strategy capable of enabling large-scale visual screening is training teachers to apply the VA test using the Snellen chart. Other authors have recommended and validated this strategy [3,6,20,21]. Based on these premises, the Visual Disorders Combat League was created in 2021 by medical students from the State University of Feira de Santana. Grounded on the university triad and focusing on extension, one of the league's objectives is to promote eye health for children lacking ophthalmological care in Feira de Santana/Bahia, and the surrounding region.

Factors associated with LVA in this study were female gender and white race. This association with female gender has been evidenced in other research [6]. No national studies associating ethnicity and LVA were found, but research in the United States showed that children who self-identified as black had worse VA [22,23]. Foreign literature also reports an association between family income and LVA [23,24], a relationship not demonstrated in this study. Further national studies are needed to better elucidate these factors.

LVA was found more frequently in subjects with excessive screen use (31.7% vs. 26.5%) compared to those without excessive use, although we did not find a statistically significant difference. However, we demonstrated an association between excessive screen use and some ophthalmological symptoms. There are still few studies on this topic in the literature, as the issue of screens is relatively recent, and the true impact on eye health is still unknown [25].

A recent meta-analysis revealed that excessive smartphone use can increase the chance of ocular symptoms such as blurred vision, as well as myopia, asthenopia, and ocular surface diseases [26]. Besides the neuropsychomotor and social benefits, restricting prolonged screen use seems to positively impact eye health, making parental involvement indispensable in monitoring and regulating excess [26-28].

This study has some limitations. In 2021, it was estimated that Feira de Santana had 16,364 children enrolled in the second to fourth grade in municipal schools [29]. Due to logistical difficulties in screening and team size limitations, we had a relatively small (n = 358) and non-probabilistic sample size. Additionally, the study's unicentric nature and the subjective method of defining screen time limit our external validity.

Despite these limitations, our study has important strengths. To our knowledge, this is the first study to assess the prevalence of LVA among public school children in our region. By focusing on a population that is often underrepresented in national data - children from public schools in socioeconomically vulnerable areas - we offer valuable insights that can help inform public health strategies. These findings highlight the need for early screening, access to eye care, and preventive measures within schools to reduce the risk of undiagnosed visual problems and prevent long-term impacts, such as amblyopia, that may become irreversible if not addressed in time.

## Conclusions

LVA was identified in approximately 30% of public school children in the interior of Bahia, yet fewer than 10% of these children were currently using glasses. Alarmingly, around 70% had never undergone an ophthalmologic evaluation. Excessive screen use emerged as a significant concern, with potential harmful effects on children’s eye health.

Our study highlights the urgent need for active visual screening programs in schools, particularly in socioeconomically vulnerable regions, to detect visual problems early and prevent long-term consequences such as amblyopia. Additionally, the findings emphasize the importance of implementing community-level strategies to improve access to ophthalmologic care and provide affordable corrective lenses. By addressing these gaps, we can help ensure that children not only achieve better visual outcomes but also have improved educational and developmental opportunities.
